# Exposure to Ultrafine Particles in the Ferroalloy Industry Using a Logbook Method

**DOI:** 10.3390/nano10122546

**Published:** 2020-12-17

**Authors:** Rikke Bramming Jørgensen, Ida Teresia Kero, Aleksander Blom, Esten Eide Grove, Kristin von Hirsch Svendsen

**Affiliations:** 1 Department of Industrial Economics and Technology Management, Norwegian University of Science and Technology, NO-7491 Trondheim, Norway; aleksblom@gmail.com (A.B.); esten_e_grove@hotmail.com (E.E.G.); kristin.svendsen@ntnu.no (K.v.H.S.); 2 Department of Materials Production and Processing, SINTEF Industry, P.O. Box. 4760, NO-7465 Trondheim, Norway; ida.kero@sintef.no

**Keywords:** ultrafine particles, logbook method, exposure, nanoparticles, ferroalloy plant, smelter, ferrosilicon

## Abstract

Background: It is difficult to assess workers’ exposure to ultrafine particles (UFP) due to the lack of personal sampling equipment available for this particle fraction. The logbook method has been proposed as a general method for exposure assessment. This method measures the time and concentration components of the time-weighted average concentration separately and could be suitable for investigation of UFP exposure. Objectives: In this study, we have assessed workers’ exposure to UFP in a ferrosilicon plant. The main tasks of the furnace workers were identified, and the logbook method was used in combination with stationary measurements of UFP taken as close to the identified task areas as possible. In order to verify the results, respirable particles were collected using stationary sampling in close proximity to the UFP measuring instrument, and personal full-shift sampling of respirable particles was performed simultaneously. Thus, exposure to respirable particles determined using the logbook method could be compared to the results of standard measurement. Methods: The particle number concentration of ultrafine particles was determined using a NanoScan SMPS. Respirable particle concentration and exposure were determined using a sampling train consisting of a pump, filter, filter cassettes, and SKC Cyclone for the respirable fraction. Attendance times for workers at each work location were registered via thorough observations made by the research team. Results: The logbook method for exposure estimation based on stationary sampling equipment made it possible to calculate UFP exposure for workers operating the furnaces at a ferrosilicon plant. The mid-size furnace and the large furnace were evaluated separately. The workers operating the largest furnace were exposed to 1.47 × 10^4^ particles/cm^3^, while workers operating the mid-size furnace were exposed to 2.06 × 10^4^ particles/cm^3^, with a mean of 1.74 × 10^4^ particles/cm^3^. Substantial contributions from the casting area, ladle transport corridor, and both tapping areas were made. Exposure to respirable particles was 2.04 mg/m^3^ (logbook); 2.26 mg/m^3^ (personal sampling) for workers operating the large-sized furnace, 3.24 mg/m^3^ (logbook); 2.44 mg/m^3^ (personal sampling) for workers operating the medium-sized furnace, and 2.57 mg/m^3^ (logbook); 2.53 mg/m^3^(personal sampling) on average of all tappers. The average ratio of these two methods’ results was 1.02, which indicates that the logbook method could be used as a substitute for personal sampling when it is not possible to perform personal sampling, at least within this industry. Conclusions: The logbook method is a useful supplement for exposure assessment of UFP, able to identify the most polluted areas of the workplace and the contribution of different work tasks to the total exposure of workers, enabling companies to take action to reduce exposure.

## 1. Introduction

It is well known that workers in the smelting industry are exposed to a variety of gases, fumes, and dust particles that may adversely affect their respiratory system. Assessment of worker exposure to respirable particles, total dust, and airborne fibers has been performed in these industries and is heavily documented [1,2,3,4,5]. The importance of airborne ultrafine particles (UFP) has received increasing amounts of attention in recent years. Hot processes in the smelting industry such as tapping, refining, and casting are sources of thermally generated fumes and are thus expected to generate UFP emissions in this industry as well. High concentrations of UFP have been observed in production facilities for silicon alloys, aluminum alloys as well as silicon carbide production plants, iron foundries and stainless-steel production facilities [6,7,8,9,10,11,12,13,14,15].

Exposure assessment of UFP is difficult and rarely performed using the methods recommended in general for workplace exposure assessments. Therefore, little is known about workers’ exposure to UFP in industrial workplaces, the existing knowledge relates mainly to UFP emissions from job activities [13,14,15,16,17,18,19,20,21,22]. Traditional workplace exposure assessments are performed via estimation of the concentrations inhaled of a specific fraction of particles in the air. Hence, sampling is performed in the breathing zone through the use of a sampling train consisting of a pump, filter, and particle size-selective sampling head, such as an aluminum cyclone for respirable particles or aerosol sampler for inhalable particles. For UFP measurement, these types of personal measurement equipment and sampling heads do not exist.

One reason for this is that a separation technique is essential in order to estimate the correct UFP concentration from an air sample that contains a large range of particles in the workplace atmosphere. Existing separation techniques include a three-step process beginning with particle charging followed by separation of charged particles in differently sized fractions, and finally quantification of the number of particles in each fraction size. The instruments used in this process often include a preconditioner, which is an impactor that removes larger particles. Different charging techniques are used; for example, the Scanning Mobility Particle Sizer (SMPS) uses either a radioactive source or an X-ray source, while other instruments, such as the Fast Mobility Particle Sizer (FMPS) and ELPI^®^, use corona charging. Separation equipment includes cascade impactors or Differential Mobility Analyzers. None of these techniques are available as sampling heads. The instruments are large, demanding in terms of power, and only suitable for stationary measurement. For the purposes of this study, a smaller, battery-operated instrument is introduced, the NanoScan SMPS from TSI, but even this instrument is not portable. It cannot be carried around by a worker for personal exposure assessment, but it can be deployed in an industrial workplace, something which is not generally possible for a conventional SMPS.

Smaller pieces of equipment, such as the Nanotracer from Philips as well as the P-Trak and CPC 3007 from TSI, are small enough to be portable and consequently may be used for personal exposure assessment. Møller et al., (2014) measured particle concentrations by fixing a Nanotracer to a belt at the hip of airport workers. The results demonstrated a strong gradient of exposure among the occupational groups, with baggage handlers exposed to average concentrations seven times as high (GM: 37 × 10^3^ particles/cm^3^) as those of indoor workers (5 × 10^3^ particles/cm^3^) [23]. However, the precision of the Nanotracer is not particularly high; its specified accuracy is said to be ±1.5 × 10^3^ particles/cm^3^. Asbach et al., (2012) compared the Nanotracer to other portable nanoparticle devices and found that special care must be taken whenever the average particle size of the aerosol exceeds the manufacturer’s specifications (20–120 nm), which may be problematic if the properties of the aerosol are completely unknown [24]. The portable TSI CPC 3007 device was used in a study by Vinzents et al., (2005) in which UFP exposure was studied among test persons bicycling in traffic. Cyclists carried the instrument in backpacks with the inlet tube placed in the breathing zone [25]. The CPC 3007 has a strictly defined upper number concentration (10^5^ particles/cm^3^), which limits the use of the instrument for workplace exposure assessment. The instrument has also been tested together with other instruments for occupational hygiene-relevant solutions, and results showed that the CPC 3007 does not display comparable results with the remaining instruments and could not be used to report UFP measurements [26]. Methods for approximative estimations of worker exposure to UFP have been tested in different ways through use of the larger instruments mentioned above. Ragde et al., (2016) have investigated exposure to UFP caused by surgical smoke in operation rooms through the use of conductive sampling tubes connecting stationary equipment to the left shoulders of investigated staff members [27]. Similar procedures were used during a simulation of cook exposure to UFP emissions, a test conducted in a laboratory [28], and for tunnel rehabilitation work [29]. Elihn et al., (2008) approximatively estimated the UFP exposure of asphalt workers by mounting the measurement equipment on a van. The van followed a paving machine at the same speed as the road was paved with the inlet tubing’s at mouth level [30]. Brand et al., (2013) studied exposure to UFP during different welding techniques by using a funnel-shaped fume hood and a sampling site placed 60 cm from the welding process; this distance was used as it represents the approximate distance between the welding process and the breathing zone of the welder [31]. All of these methods are designed specifically for certain operations and cannot easily be transposed to other workplaces, such as metal production facilities. In addition, extensive use of conductive tubing should be avoided, and the tubes used should be as short as possible to avoid particle losses [32,33].

Another approach to exposure measurements is the logbook method [34] proposed by Olsen in 1994. The logbook method measures the time and concentration components of the time-weighted average concentration separately. It consists of process log keeping and process measurements, and only one process is performed during each sampling period [34]. This approach is mentioned in the workplace exposure standard NS-EN 689:2018 as one of the measurement methods for testing compliance with eight-hour OELV when workplace factors are not constant during the work shift; in NS-EN 689, it is described as multiple exposure scenarios throughout the whole shift [35]. Although the method is described as useful for personal measurements, we wanted to see if this method could be applied for stationary measurement use in combination with traditional sampling. Focusing on one work task during each sampling period makes it possible to use stationary equipment.

The purpose of this study is to perform an assessment of workers’ exposure to UFP in a ferrosilicon plant. Based on knowledge gained from previous exposure studies, the workers expected to face the highest amounts of exposure are tappers [36]. The main work tasks for the tappers were identified and the logbook method was utilized in combination with stationary measurements of UFP through the use of a NanoScan SMPS instrument as close to the identified work tasks as possible. In order to verify the results, respirable particles were collected through stationary sampling close to the UFP measuring instrument, and personal full-shift sampling of respirable particles was performed simultaneously. Thus, exposure to respirable particles found using the logbook method could be compared to the results of standard measurement.

### Ferrosilicon Alloy Production

Ferroalloys are iron-based alloys with one or more added alloying elements. Bulk ferroalloys include many different alloys, including ferrochromium, ferromanganese, ferronickel, and ferrosilicon [37].

**Figure 1:**
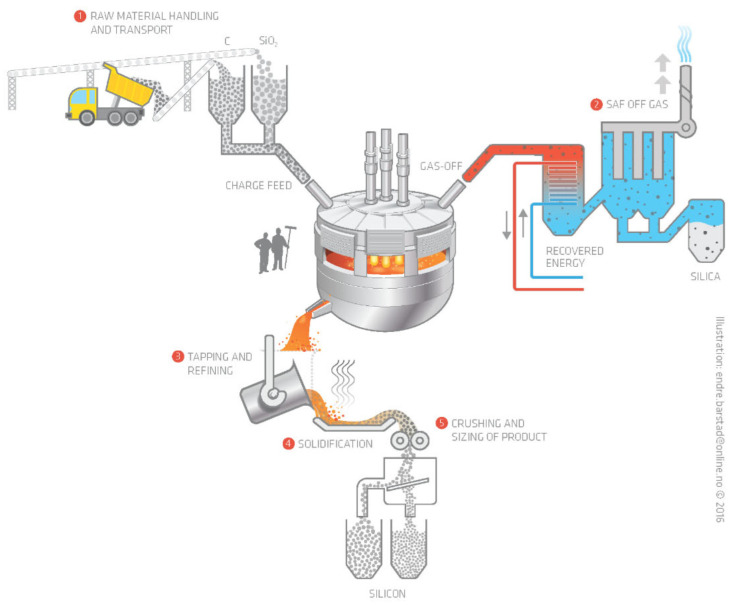
Overview of processes in a typical smelter for ferrosilicon alloys. Reproduced from [6] with permission from Springer Link 2017.

The term ferrosilicon (FeSi) denotes a group of ferroalloys that typically contain 65–90% silicon (Si). The core processing unit for ferrosilicon production is the submerged arc furnace. The primary raw material is quartz, which is fed to the furnace together with additions of iron ore (or scrap). The reductants include coal, charcoal, wood chips, and sometimes coke. The high-temperature process continuously consumes the raw materials, the reductants, and the three carbon-based electrodes. At approximately 2000 °C, the carbon reacts with the oxygen in the quartz and iron ore so that a liquid metal forms and can be tapped from the furnace. After tapping, the ferrosilicon is usually refined, cast, and solidified. Once the metal is cooled, it is crushed and sieved into pieces of variable sizes. Figure 1 shows a process overview from a typical smelter [6].

## 2. Methods

### 2.1. Process Description

The company conducted exposure assessments of respirable particles and total dust over a time period of five years. Based on these exposure assessments, the workers performing furnace tapping (henceforth referred to as tappers) were identified as the workers with the highest exposure to UFP and therefore were the group selected to be investigated in this study.

At the plant studied, the primary tasks of the tappers are:

- tapping the liquid alloy from the furnace to the ladles—performed in a location called the tapping area
- transporting the ladles from the furnace to the casting area—performed in a location called the ladle transport corridor
- casting, or pouring the liquid alloy into sand beds where it solidifies—performed in a location called the casting area
- monitoring the furnace process from a control room with video monitors and other process data collected on computer screens—performed in a location called the control room

The plant holds three furnaces, the two largest of which were selected for this study, as the space conditions surrounding the smallest furnace did not allow us to carry out measurements there. The sizes of these furnaces are 42 MW and 45 MW. The large furnace was operated continuously by two workers on each shift. The mid-sized furnace was operated by one worker during each shift. Henceforth, the two job-groups will be referred to as work groups L and M, respectively.

### 2.2. Logbook Method

Walkthrough surveys of the plant were performed, and information on jobs and tasks was collected. Based on the information, four job-locations were defined as important for determining exposure levels: the tapping area, the casting area, the ladle transport corridor, and the control room, as described in Table 1. Each job-location is dominated by one job-task, or job-process, according to the terminology of Olsen [34].

Attendance times for the workers at each work location were registered using thorough observations made by the research team. Each of the researchers followed one worker at a time and logged all activities the worker performed each day. Detailed descriptions of each employee’s workday were obtained, divided into periods the worker spent at each of the four locations and the subtasks performed during each period (blowing of oxygen, closing of tap holes, etc.).

The measuring equipment was placed as close as possible to the worker and the job-task. UFP measurements were performed continuously during the measurement period at a rate of one minute per sample. Based on the registered attendance time at the specific location, the UFP concentrations (C_1_, C_2_, etc.) relevant for calculation of the exposure were recalculated from the measured dataset. For respirable particles, the exposure was measured as the concentration of the respirable fraction of aerosols in the air only during the attendance time. The respirable particles were sampled through the use of a sampling train consisting of a pump, filter, and cyclone. The equipment was placed stationary close to the UFP equipment and switched on or off to reflect the presence of the worker. The sampled respirable fraction was thus representative of the concentration during the attendance time.

In total 25 attendance time loggings were done, of them 18 loggings for Workgroup L and 7 for workgroup M. 13 stationary full workday measurements of UFP were performed, 3 measurements in the ladle transport corridor, control room, casting area and tapping area for Workgroup M, 4 full workday measurements in tapping area for Workgroup L.

Two stationary full-day (8 h) samplings of respirable particle was planned for each of the corresponding UFP full workday measurement. Six samplings of respirable particles were performed as 8-h sampling, but due to high concentrations of particles at some locations, the sampling intervals had to be shortened. The remaining samplings were performed as 4-h samplings of respirable particles. The results are then recalculated to 8-h results, made up of 4-h samplings periods measured the same day.

### 2.3. Personal Samples of Respirable Particles

Exposure was measured as the concentration of the respirable fraction of aerosols in the air by mounting the equipment in the breathing zone of the worker. The pump was placed in a rucksack, and tubing was fastened to the rucksack using adhesive tape. Sampling was performed throughout the entire workday.

Four full workday measurements were planned for both workgroups. Four full workdays measurements were performed for Workgroup L, three full workday measurements for tappers in Workgroup M. Two of the measurements were performed as 8-h measurements, the remaining as two half-shift (4 h) sampling periods.

### 2.4. Air Measurement Methods

All air measurements and samplings were performed during a 12-day period in March 2019. Two NanoScan SMPS (Model 3910, TSI, Shoreview, MN, USA) units were used to continuously measure UFP in this study. The NanoScan SMPS measures the number concentration of submicrometer particles (including ultrafine particles) and the particle-size distribution in the size range 10–420 nm in 13 fractions. Principle particle sizing was performed using a unipolar charger, and the classification was based on the electrical mobility in a radial differential mobility analyzer (rDMA). The quantification of particles was performed using a condensation particle counter (CPC) integrated into the instrument. Particle classification and counting were performed simultaneously with a time resolution of one minute. The measurement principle is described in detail elsewhere [26]. Before entering the measurement units, the aerosol flow passes a cyclone with a cut point (D_50_) of 550 nm to remove larger particles. The instrument operates at a flow rate of 0.75 L/min. The CPC is isopropanol-based, and reflooding is needed during a work shift of 8 h. The instrument is battery-operated and the battery lasts for approximately 6 h. In order to secure proper measurements, all measurements stopped after 4 h of operation for reflooding with isopropanol and charging of batteries. The instruments were covered with a heat-protecting blanket to protect against metal splashing, sparks, and heat radiation. A 0.6 m flexible conductive silicone tube was used to make sure that air sampling was performed outside of the heat-protected area.

For the sampling of respirable particles, pre-weighed glass fiber filters (Gelman Sciences type A/E 37 mm) were used. The filters were placed in closed-face Millipore cassettes mounted with SKC aluminum cyclones. The sampling head was connected to a pump (AirChek 3000, SKC Ltd., Dorset, England) with an airflow of 2.5 L/min. The pump flow was controlled before and after each sampling period. 12 blind filters were used as controls to correct for handling and environmental factors. The masses of all air filters were measured using a scale, the Mettler AE 163 (Mettler Instruments AG, Greifensee, Switzerland), with a limit of detection (LOD) of 0.05 mg.

The sampling period was originally planned to be 8 h long, but due to high concentrations of particles at some locations, the sampling intervals had to be shortened. To prevent overloading of filters, each shift was divided into two half-shift (4-h) sampling periods.

### 2.5. Data Analysis

The UFP measurements were performed using the NanoScan Manager software (TSI, version 1.0.0.19). The particle number concentration within the size range 10–420 nm was measured in 13 channels. The number concentration of nanoparticles was calculated in Microsoft Excel as the sum of particles of the first 8 channels, resulting in a particle concentration in the range 9.8–100.0 nm. The unit of the particle number concentration is the number of particles per cm^3^ and the notation is #/cm^3^_._ UFP measurements were performed continuously during the measurement period at a rate of one minute per sample. Based on the registered attendance time at the specific location, the UFP concentrations (C_1_, C_2_, etc.) relevant for calculation of the exposure were recalculated from the measured dataset. The results are calculated as arithmetic mean (AM) with corresponding standard deviation (SD).

Attendance time was calculated as the percentage of workday spend at each location based on the logbook for each worker. In addition, minimum and maximum percentage of workday spend at each location was calculated.

The time-weighted average concentration was calculated as [38]:

(1) $$TWAC=\frac{1}{8\;hour}\;\sum_{x=i}^{m_{j}}\Delta t_{jxp}C_{jxp}$$

where

$\Delta t_{xp}$: the average duration of period *p* during the time in which the specific job-task is performed
$C_{xp}$: average concentration in that job-task period
m: number of job-task periods

## 3. Results

The research team performed 25 registrations of the workers’ activities at each job-location during the workdays. The logging was performed on seven different days during a period of 12 days and represented day shifts on both weekdays and weekends. The results are shown in Table 1:

For Work Group L, the day-to-day variation in percentage distribution between the work locations was high. The more time the workers spent in the control room per day, the lower the exposure was expected to be. The minimum and maximum percentages of the workday spent at each location are included in Table 1.

The concentrations of UFP and respirable particles were measured at all job-locations: the tapping area for both the mid-size and large furnace, the casting area, the ladle transport corridor, and the control room. Each job location represents one job-task, and the measurements were performed only while the worker performed this specific job-task. The results are displayed in Table 2:

The exposure of the workers was calculated using Equation (1). The attendance times are given in Table 1, and the concentrations for UFP and respirable particles are given in Table 2. Calculated logbook results for ultrafine particles are shown in Table 3.

Table 4 shows the results of the personal sampling and the calculated logbook results for respirable particle exposure. Results are given for work groups M and L as well as the mean for all tappers. The logbook results are calculated based on the attendance times given in Table 1 and the job-location based measurements given in Table 2.

As seen in Table 4, the ratios between logbook method results and personal sampling are between 0.75 and 1.11, with an average of 1.02 as the mean for all tappers.

## 4. Discussion

In order to verify the methods used, the ratios between logbook results and personal sampling were calculated for respirable particles. These ratios amounted to 0.75 for work group M and 1.11 for work group L, with an average of 1.02 for all tappers. This good agreement between the respirable particle measurements indicates that the logbook method based on stationary measurements is suitable for estimating the exposure of tappers to respirable particles. The limitations of the use of stationary measurement equipment instead of measurements performed in breathing zones are well known. The ratios of personal sampling to the logbook method for respirable particles indicate however, that the exposure levels found by stationary measurement were close to the exposure levels found using personal sampling, at least in this case. Whether this good agreement between the two types of measurements can be transferred to the exposure to ultrafine particles is not clear. Pollution sources could differ, and particle air movement and settlement could differ between respirable particles and ultrafine particles as well. Respirable particles are, however, the particle fraction closest to the size of ultrafine particles and at the present moment, it may be the best method available due to the lack of personal sampling equipment for UFP. Jensen et al., (1995) assessed long-term styrene exposure, and the results showed that the logbook method compared well with earlier published results on traditional personal sampling for styrene exposure [39]. The results by Jensen et al. confirm the applicability of the method on a general basis.

The method demonstrated here is useful for measuring exposure to ultrafine fractions in field. The NanoScan SMPS was operated through the use of batteries and isopropanol loading of the wick, which made it possible to place the equipment without considering proximity to a power supply. This method also avoids the uncertainty introduced by the use of long sampling tubes [31,32]. The measuring equipment was placed as close to the workers and work processes as possible. This is an ordinary restriction in this type of industry with large power installations and high temperatures. Preliminary measurements were performed with handheld equipment in order to ensure that the concentrations at the measurement equipment were close to the concentrations at the worker positions. Ratios between personal sampling and logbook results close to 1 indicate that the placement was acceptable, at least with respect to the respirable particles. For respirable particles, a sampling train exists. A portion of the personal samples performed was, however, rejected. The temperature conditions for the cassettes containing the glass fiber filters were too high, causing some cassettes to melt. Additionally, pump operations were somewhat unstable during the measurement campaign. This problem was avoided using stationary placement.

Full-day (8-h TWA) worker exposure to UFP was 1.47 × 10^4^ particles/cm^3^ for work group L, and 2.06 × 10^4^ particles/cm^3^ for work group M, with a mean of 1.74 × 10^4^ particles/cm^3^. This is, to our knowledge, the first estimate of worker exposure to UFP in this industry. Earlier studies have only examined process concentrations. Kero and Jørgensen (2016) found tapping area concentrations in the range 2.16 × 10^4^–7.06 × 10^4^ particles/cm^3^, measured 15 m from the ladles [7]. The process-based results of this study (Table 2) are lower than that, even though the instruments were placed closer to the furnaces than in the previous study.

No occupational exposure limits (OELs) are available for UFP. However, nano reference values (NRV) have been proposed for nanoparticles, with a special focus on manufactured nanoparticles [40,41,42]. Since UFP may be regarded as a subgroup of nanoparticles, NRV values can be used here. The NRV values relate to the density, form, and biopersistence of particles. In this case, Class 2 (nanomaterials with densities below 6000 kg/m^3^) is relevant, with an NRV of 4.00 × 10^4^ particles/cm^3^ given as an 8-h TWA value. No safety factors have been established for the evaluation of NRV values, but the uncertainty in quantification of exposure has been evaluated using NS-EN 689: Workplace exposure—Measurement of exposure by inhalation to chemical agents—Strategy for testing compliance with occupational exposure limit values [35]. NS-EN 689 does not relate to the logbook method but is rather a strict interpretation of the standard. According to the *preliminary test*, as mentioned in NS-EN 689, compliance is achieved if all results in this case are below 0.1 OEL (because three or four individual measurements were behind each process based average mean concentration). Under this interpretation, the NRV values have been exceeded for tappers at both the large and the mid-sized furnaces as well as the mean values of both groups.

The logbook method is based on separate measures of the time and concentration components of the time-weighted average concentration. This makes it possible to evaluate the contributions from different processes or work-tasks to the exposure and the variation between them. Similar principles were successfully used in studies of the ultrafine particle exposure to which a population is exposure on individual level during 24 h periods [43,44]. One of the strengths of this study is that the research team conducted detailed registrations of the attendance time of the worker at each work location and simultaneously noted all special activities or sources of pollution. This means that the log contains information more detailed information than previous studies of this type, where the employee were asked to keep a logbook were each process was described by the name used in the company [23,27,28], or, in population studies where the participants were asked to report in a diary when their microenvironment change [32,33]. As mentioned in the introduction, the tappers were selected for this study based on internal reports from the company health service, which identified this group of workers the one faced with the highest exposure to respirable particles. Additionally, tappers work close to hot processes in the tapping area, which are known to produce UFP, and the research team thus expected to find the largest concentrations of UFP in the tapping area [6,45]. However, this was not the case in this study, as both the casting area and the ladle transport corridor displayed higher AM values than the tapping areas. The ladle transport corridor is an open area with various equipment in the ceiling for transportation of the ladles. Emissions of particles from the ladles as they are transported are predictable; however, this only occurs in limited periods and cannot be the only explanation for the high concentrations of both UFP and respirable particles in that area.

The ventilation in the plant is divided into two parts; the active ventilation consists of large ventilation hoods directly above the furnaces, including the tap holes. In the casting area, there is no active ventilation. Instead, there is a large opening in the roof above the casting area. The alloy temperature while casting is typically above 1500 °C. Thus, the heat from the metal itself creates a significant chimney effect in the direction of the ceiling. Large gates into the hall supply fresh air to the area. The chimney effect above the casting bed is mimicked by other hot processes in the building. Some of these are continuous, while others are batch-wise. Some always occur in the same place, while others may be mobile (such as the ladle refining and transport processes). This creates strong air movements inside the building, and the opening and closing of large gates for vehicle transport and other purposes lay the foundation for a dynamically changing air flow pattern within the building. The phenomenon is commonly referred to as hall wind and is known to be able to offset even the strongest of active ventilation installations in this type of industry [46]. It is likely that this kind of hall wind may be almost as important for the prediction of particulate matter concentrations in a building as proximity to the sources themselves.

Attendance times for workers at each work location were registered via thorough observations made by the research team. This made it possible to identify differences in worker exposure levels by denoting the amount of time spent in the most polluted areas. Table 3 shows that the best case for workers at the large furnace was 1.11 × 10^4^ particles/cm^3^, while the worst case was 1.51 × 10^4^ particles/cm^3^. Even the worst-case exposure is lower than the exposure at the mid-sized furnace. This difference is primarily due to attendance times at the tapping area. The mid-sized furnace was operated by one worker at the time (this worker also operates the small-sized oven), while the large furnace was operated by two workers in cooperation. This indicates that planning work tasks and sharing responsibility for the operation of furnaces could be used to lower future exposure levels of plant operators.

When comparing the activity logs to the concentration versus time curves, some of the peak exposures were associated with local sources, in this case specific tap-hole operations like the use of oxygen lance and stoking iron to increase the flow from the tap hole, which in is accordance with earlier findings in this industry [7]. These operations typically produced concentration peaks in the tapping area. In the casting area, various operations produced peaks, such as the removal of bottom slag from the ladles; additionally, ladle transport coincides in time with high concentrations elsewhere. This type of exposure analysis was demanded by Koponen et al., (2015) in their study of worker exposure in paint factories. This study investigated exposure in areas outside the predefined work-task–generated substantial contribution to exposure [47].

The workers’ exposure levels to respirable particles were 2.53 mg/m^3^ on average for all tappers measured by personal sampling. These results were from days described by the tappers as “quiet” days, with plenty of time spent in the control room. Earlier unpublished results have displayed higher levels of exposure, indicating that efforts to reduce exposure have been successful, especially at the large furnace. Compared to measurements of total dust at comparable smelters of 2.3 mg/m^3^ [48] and 4.8 mg/m^3^ from the years 1974 to 1979, 3.3 mg/m^3^ from 1980 to 1985, and 3.4 mg/m^3^ from 1986 to 1990 [49], the results of this study were similar. The OEL was 5 mg/m^3^, and the results do not show compliance with the OEL, according to the statistical methods in NS-EN 689, which are reflected by the extensive use of personal protection equipment by all workers.

Previously, UFP measurements in this industry have been performed as stationary measurements only and without attempts to estimate worker exposure [6,7,8,9]. No measurement of UFP at the actual smelter has been performed previously. Kero et al., (2013) measured the UFP of the tapping process at a ferrosilicon smelter with equivalent processes throughout three hours of continuous tapping [8]. The results of this study showed that 57.7% of the number concentrations of particle emissions were below 0.1 µm and classified as UFP. Kero and Jørgensen (2016) studied the emissions of tapping in a silicon alloy production plant with the same type of furnaces and found considerable emissions of UFP produced during tapping [7]. The location of the measurements to Kero and Jørgensen could be compared directly to the furnace tapping area measurements in this study. Kero and Jørgensen found arithmetic mean concentrations of UFP in the range 2.18 × 10^4^–7.06 × 10^4^ particles/cm^3^ which is directedly comparable with the results in this study.

Only a few comparable exposure assessments of UFP during 8-h working day exists and none of them were performed in this industry. Elihn et al. found an average particle concentration during paving at 3.4 × 10^4^ particles/cm^3^, with large differences between different asphalt paving activities [20]. Cheng et al. found an average concentration in the casting area in an iron foundry 2.07 × 10^4^–2.82 × 10^5^ particles/cm^3^ with an mean value at 7.06 × 10^4^ particles/cm^3^ [22]. Studies of area specific concentrations of UFP related to different work tasks are performed in stainless steel production [14]; and in grey iron foundry [15]. Evans et al. performed multiple measurements in a grey iron foundry during different seasons. Particle concentrations were the lowest during the summer (7.01 × 10^4^–1.68 × 10^5^ particles/cm^3^). In the wintertime, concentrations varied from 2.09 × 10^5^ to 2.39 × 10^5^ particles/cm^3^. The highest number concentrations were measured in the alloy melting deck (1.6 × 10^6^ particles/cm^3^) [16]. Järvela et al. found particle number concentrations in ferrochromium and stainless steel production from 5.8 × 10^4^ particles/cm^3^ to 6.62 × 10^5^ particles/cm^3^ in the production areas [14]. Welding is another example of a relevant work-task where exposure is studied [29,30,50] the duration of welding tasks is however, shorter in time and thus les comparable in this context. Both Evans et al., (2017) and Järvela et al., (2016) report their results as total concentration measured by SMPS instruments, which could be one explanation for higher concentrations in these studies [14,15], another explanation could be real differences between the industries. Further studies of the particle size distribution in the different industries is recommended in order to explain the observed differences.

As pointed out by Olsen et al. [34] the logbook method includes a detailed analysis of exposure by which processes can be ranked after their contributions to workers’ exposure. This make it possible for the production plant to investigate or prepare preventive measures against all processes with high contribution to worker exposure. The same benefits are found by including attendance hours in exposure studies within public health perspective [43]. Focusing processes in the production plant allow the company to implement measures in accordance with the principles of the hierarchy of measures [51,52] focusing engineering control before administrative controls and personal protective equipment.

## 5. Conclusions

The logbook method for exposure estimation based on stationary sampling equipment made it possible to calculate UFP exposure for workers operating furnaces at a ferrosilicon plant. The workers operating the largest furnace were exposed to 1.47 × 10^4^ particles/cm^3^, while workers operating the mid-size furnace were exposed to 2.06 × 10^4^ particles/cm^3^, with a mean of 1.74 particles/cm^3^. For practical and safety reasons, no measurements could be made at the smallest furnace of the plant.

A comparative study with parallel sampling of respirable particles made it possible to compare the logbook method to traditional personal sampling of respirable particles for the same groups of furnace operators. Exposure to respirable particles was 2.04 mg/m^3^ (logbook) compared to 2.26 mg/m^3^ (personal sampling) for workers operating the large size furnace, 3.25 mg/m^3^ (logbook) compared to 2.44 mg/m^3^ (personal sampling) for workers operating the medium-sized furnace, and 2.53 mg/m^3^ compared to 2.57 mg/m^3^ on average. The ratio on average was 1.02, which indicates that the logbook results could be used as a substitute for personal sampling when it is not possible to perform personal sampling, at least in this industry.

The advantage of the logbook method is that it allows researchers to identify which processes and areas of the plant primarily contribute to exposure and provides subsequent opportunities to focus on prevention measures—in this case, the ladle transport corridor and the casting area.

## Figures and Tables

**Table 1:** Percentage of workday spend at each location for the tapping hall workers alongside minimum and maximum percentage of workday spend at each location. Note that casting area and ladle transport corridor were common areas used for both furnaces.

	Work Group M %	Work Group L %	Mean for All Tappers %
Tapping area	48 (26–56)	25 (8–53)	36
Casting area	7 (3–11)	8 (3–21)	8
Ladle transport corridor	16 (6–26)	15 (8–25)	15
Control room	29 (18–46)	52 (27–76)	41

**Table 2:** Results of all stationary job-location measurements. Ladle transport corridor and casting area are common areas. Control room measurements have been taken only in control room close to the large furnace. AM: arithmetic mean, SD: standard deviation.

		UFP (#/cm^3^)	Resp. (mg/m^3^)
Mid-size furnace tapping area	11.3	3.52 × 10^4^	-
	13.3	1.86 × 10^4^	2.49
	14.3	2.23 × 10^4^	3.49
	AM	2.53 × 10^4^	2.99
	SD	8.72 × 10^3^	0.71
Large furnace tapping area	8.3	2.85 × 10^4^	5.13
	9.3	2.96 × 10^4^	3.17
	10.3	1.53 × 10^4^	4.75
	12.3	1.27 × 10^4^	6.93
	AM	2.15 × 10^4^	5.00
	SD	8.77 × 10^3^	1.54
Casting area	8.3	6.58 × 10^4^	1.34
	9.3	1.85 × 10^4^	2.24
	10.3	2.01 × 10^4^	3.13
	AM	3.48 × 10^4^	2.23
	SD	2.69 × 10^4^	0.89
Ladle transport corridor	11.3	4.50 × 10^4^	-
	12.3	3.70 × 10^4^	5.17
	13.3	2.35 × 10^4^	4.69
	14.3	-	5.15
	AM	3.52 × 10^4^	5.00
	SD	1.09 × 10^4^	0.27
Control room	8.3	6.28 × 10^3^	0.20
	9.3	3.43 × 10^2^	0.10
	10.3	5.08 × 10^2^	0.15
	AM	2.38 × 10^3^	0.15
	SD	3.38 × 10^3^	0.05

**Table 3:** Logbook results for UFP exposure.

	Exposure to UFP (particles/cm^3^)
Work group M	2.06 × 10^4^
Work group L	1.47 × 10^4^
Average for all tappers	1.74 × 10^4^
“Best case” for work group L	1.11 × 10^4^
“Worst case” for work group L	1.51 × 10^4^

**Table 4:** Logbook results for respirable particle exposure, personal sampling, and ratio between the two types of results.

	Work Group M	Work Group L	Average of All Tappers *
	Conc. (mg/m^3^)	Conc. (mg/m^3^)	Conc. (mg/m^3^)
Personal sampling	3.15 *	1.30 *	
	2.75 *	1.04 *	
	3.82 *	3.98 *	
AM Personal sampling	3.24	2.04	2.53
logbook method	2.44	2.26	2.57
Ratio	0.75	1.11	1.02

* average of all tappers are calculated from all marked values.
